# Choosing Wisely in oncology in Latin America: what SLACOM does not recommend in the care of cancer patients in Latin America: Ten essential recommendations to avoid harmful onclogy procedures in Latin America

**DOI:** 10.3332/ecancer.2024.1691

**Published:** 2024-04-09

**Authors:** Julia Ismael, Eugenia Esandi, Gerardo Arroyo, Sergio Becerra, Suyapa Bejarano, Carlos Castro, Claudia Enrique, Cinthia Gauna, Francisco Gutiérrez-Delgado, Ernesto Gil Deza, Karin Kopitowsky, Daniel Lewi, Bettina Muller, Raul Murillo, Alicia Pomata, Jorge Puyol, Gabriela Quintanilla, Silvana Rompato, Luiz Santini, Tatiana Vidaurre, Angela Solano, Daniel Campos, Eduardo Cazap

**Affiliations:** 1 GEDYT S.A., Juncal 2345, C1125ABE, Buenos Aires, Argentina; 2 Academia Nacional de Medicina J.A. Pacheco de Melo 3081, C1425AUM, Buenos Aires, Argentina; 3 CeDIT, Santiago del Estero 1415, A4400 Salta, Argentina; 4 Fundación CARE, Bucarest 17, Of.81, ZC7510031, Providencia, Santiago de Chile, Chile; 5 Liga Contra el Cáncer, 8 calle 11 avenida SO No. 51, ZC21101, San Pedro Sula, Honduras; 6 Liga Colombiana Contra el Cancer, Carrera 12A No. 77 – 34 ZC110911, Bogotá, D.C., Colombia; 7 Provincial Cancer Institute, Entre Ríos Montiel 1274, E3104BIO, Paraná, Entre Ríos, Argentina; 8 Sociedad Paraguaya de Oncologia Medica, Las Residentas 744, Fdo de la Mora, ZC110306, Asunción, Paraguay; 9 ELO-CEPREC, San Pedro 136, Col. La Gloria ZC29064, Tuxtla Gutiérrez, Chiapas, Mexico; 10 Henry Moore Institute, Gral Las Heras 1917, CP 1602, Florida, Buenos Aires, Argentina; 11 Argentine Society of Family Medicine, Fragata Sarmiento 885, C1405API, Buenos Aires, Argentina; 12 Hospital Fernadez- Medicus, Av. Cerviño 3356, C1425AGP, Buenos Aires, Argentina; 13 Grupo Oncológico Cooperativo Chileno de Investigación, José Manuel Infante 125, Providencia, Santiago, Chile; 14 Centro Javeriano de Oncología, Tv. 5 #412, ZC110911, Bogotá, Colombia; 15 Programa Nacional de Control del Cancer, Rogelio Benitez 912 v1 casi Antolin Irala, ZC060404, Asunción, Paraguay; 16 Argentine Society of Cancerology, Tinogasta 2863 4 A, C1417EHI, Buenos Aires, Argentina; 17 National University of Rosario, Maipú 1065, S2000CGK Rosario, Santa Fe, Argentina; 18 Asoc. Argentina de Oncología Clinica, Moreno 150, P3600KAD, Formosa, Argentina; 19 Former director, INCA, Rua André Cavalcanti, 37 - 2º andar, Rio de Janeiro, RJ 20.231-050, Brazil; 20 Instituto Nacional de Enfermedades Neoplásicas, Av. Angamos Este 2520, ZC15038, Surquillo, Peru; 21 Centro de Educacion Médica e Investigaciones Clínicas - HUSS, Avda. Galván 4102, C1431FWO, CABA, Argentina; 22 Sociedad Latinoamericana y del Caribe de Oncología Medica (SLACOM) Av. Córdoba 2415, 5º Piso, C1120AAG, Buenos Aires, Argentina, and ecancer; a https://orcid.org/0000-0001-9513-0681; b https://orcid.org/0000-0002-8589-5725; c https://orcid.org/0000-0002-6342-3663

**Keywords:** Choosing Wisely, Eligiendo Sabiamente, oncology, Latin America

## Abstract

Choosing Wisely is an initiative by the American Board of Internal Medicine (ABIM) and ABIM Foundation to deter unnecessary medical treatments and procedures. Faced with the burden of modern technologies and treatments, it is crucial to identify practices lacking value in daily care. The Latin American and Caribbean Society (SLACOM), comprising cancer control experts, deems it vital to tailor this initiative for enhancing cancer care in the region. Through a modified DELPHI methodology involving two rounds of electronic questionnaires and a hybrid meeting to discuss key points of contention, ten essential recommendations were identified and prioritised to avoid harmful oncology procedures in our region. These consensus-based recommendations, contextualised for Latin America, have been compiled and shared to benefit patients. The Scientific Committee, consisting of prominent oncologists and health experts, collaborates remotely to drive this project forward.

## Introduction

Cancer is a devastating disease that affects millions of people worldwide. With the advancement of medical technology, cancer treatments are becoming increasingly complex and varied. Today, patients have a wide range of options available to them and it is essential to make decisions based on scientific evidence to maximize outcomes and minimize risks. Choosing wisely (CW) in cancer is critical to achieving the best possible outcomes and quality of life [1–3].

This disease is a major public health problem in Latin America and the Caribbean (LAC). According to the International Agency for Research on Cancer, the age-standardised cancer rate in LAC is one of the highest in the world [4]. In the region, cancer is the second leading cause of death after cardiovascular diseases. Breast, cervical and prostate cancer are the three most common cancers in the region [5].

To reduce the burden of cancer, governments and other stakeholders in LAC have implemented policies and programs to reduce risk factors and increase access to diagnosis and treatment. These include increasing public awareness of risk factors, strengthening health systems, providing access to high-quality health services and developing new treatments and technologies.

However, government efforts in cancer control focus more on diagnosis and treatments [6].

The absence of prevention policies is associated with late diagnosis and the consequent lack of efficiency in terms of cost-effectiveness [7].

The overload of new technologies and treatments makes it essential to urgently define which diagnostic procedures or treatments are unnecessary or of little or no value in daily clinical practice.

‘CW’ is an initiative created by the American Board of Internal Medicine (ABIM)–ABIM Foundation in 2012 to avoid unnecessary medical tests, treatments and procedures.

The mission of ‘CW’ is to promote conversations between physicians and patients by helping patients choose care that is supported by evidence by avoiding repeating tests or procedures already received, free from harm and truly necessary [7].

So far there are initiatives in North America (Canada and ASCO [8]), some European countries, and more recently Africa and India.

SLACOM (LAC Society) brings together cancer experts in a comprehensive strategy that encompasses education, research, prevention, diagnosis, treatment, palliative care, access to morphine and end-of-life support. It works in close collaboration with health systems and public policies in Latin America. It is an integrated network of experts, institutions and civil society to reduce the incidence and improve the cure of cancer [9].

This is why we consider it a priority to develop a CW initiative for our region, which urgently needs to optimise existing health systems and improve the quality of cancer care for the benefit of patients and the health systems themselves.

The objective of this study is to identify practices and/or technologies of low or no value in the prevention, screening, diagnosis, treatment, and/or rehabilitation of oncologic diseases that could be subject to ‘de-implementation’ in the context of LAC countries. We will call them ‘recommendations not to do’ (RecNH).

## Methodology

**Figure 1.**
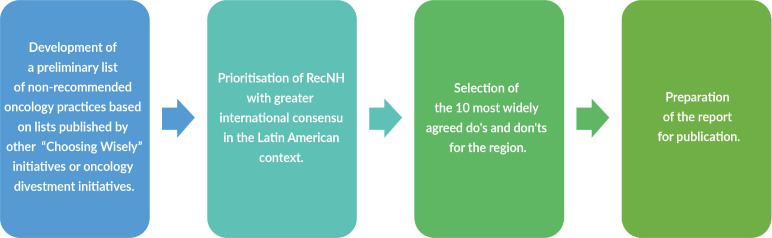
Study stages.

• The study was conducted in sequential stages (Figure 1)*.*

**1. Preparation of the preliminary list of RecNH**

**1.1.** Initially, a comprehensive review of the CW and other oncology divestment initiatives was conducted (Table 1).

**1.2.** Based on this review, a first list of ‘ RecNH’ proposed by each of these initiatives was created.

**1.3.** A mapping of the RecNH in the different lists was carried out to identify those recommendations proposed by more than one initiative.

**1.4.** A second list was drawn up with RecNH with a higher degree of consensus, i.e., those supported by multiple initiatives.

◦ **Prioritisation criteria**

- Magnitude of the problem (prevalence of the pathology).

- Variability in clinical practice.

- Aspects related to equity in access to medical care in different socioeconomic levels and countries.

- Appropriate time to treat the disease (e.g., year of colon cancer screening).

◦ **Discrepancy resolution and final selection**

- To resolve differences in the prioritisation and type of cancer, an initial consensus methodology was used. Thus, prioritisation criteria were established and agreed upon in a clear, open and organised manner, and the thematic scope was delimited.

- After consolidating a first list based on the review of evidence from multiple ‘CW’ initiatives in oncology and previous work in Argentina, 97 recommendations were identified.

- These were consolidated when their content was similar or identical.

- Subsequently, two criteria were applied to make a new selection:

1. Recommendations do not make proposals for more than one initiative (54 recommendations, reduced to 17).

2. Recommendations not proposed by a single initiative, but endorsed by multiple professional associations (*n* = 20).

- In summary, a final set of 37 Do Not Do Recommendations was obtained. These were submitted to voting by means of an online form under the Delphi methodology in 2 consecutive rounds to select the 10 don’ts with the highest degree of agreement among the experts.

**3. Prioritisation of RecNH with greater International Consensus in the Latin American Context**

**2.1. Formation of the panel of experts**: The participation of a representative of the scientific societies of different countries related to the implementation of the RecNH identified was sought. The participation of the societies of oncology, radiotherapy, surgery, palliative care, mastology and urology was considered essential. The final list of participating scientific societies was reviewed and agreed upon with the members of the Latin American panel, including experts from eight countries in the region (Table 2).

**2.2. Design of instruments for the consensus panel**: Specific instruments were developed for the Consensus Panel, as well as other elements such as letters and models of invitation to participate. These contained details on the introduction of the project, methodology, meeting dates and forms of participation.

**Expert panel consultation**: Two rounds of expert panel consultation were conducted. Each expert was asked to prioritise the recommendations based on criteria such as the burden or health impact of the disease, the risks associated with the use of the technology or practice, the ineffectiveness compared to other alternatives, the cost associated with the use of the technology, the potential impact on equity and the length and quality of life of patients.

The percentage of affirmative responses for each recommendation was estimated and the first ten recommendations with the highest percentages of acceptance were selected to form the final list.

This methodology has made it possible to identify and prioritise RecNH of relevance for improving cancer care, ensuring that the voice of Latin American experts is reflected in the final selection of recommendations.

## Results

**1. Identification of oncologic practices not recommended**

• **Source selection process**

- Start with an exhaustive search using key terms and Boolean criteria in databases, looking for the intersection of ‘CW’ and cancer-related terms with the following keywords: (choosing [All Fields] AND wisely [All Fields]) AND (‘neoplasms’[MeSH Terms*] OR ‘neoplasms’[All Fields] OR ‘cancer’[All Fields]) *MeSH Terms: Medical Subject Headings

• **Screening and selection of relevant initiatives**

- Meticulous review of various oncology divestment initiatives that included lists of ‘Do Not Do’ recommendations.

- Prioritisation of those linked to ‘CW’ and those coming from LAC countries, to ensure regional relevance.

- Eight initiatives were identified, with seven CW initiatives in countries such as the United States, Canada, Australia, Italy, the Netherlands, India and Africa, and one from Argentina (2019–2021).

• **Broad spectrum of identified practices**

- A total of 74 ill-advised recommendations were identified through this rigorous process.

- Some 60% of these recommendations are related to prevention, diagnosis, treatment and rehabilitation practices in patients with different stages of cancer, including advanced, recurrent and metastatic.

- The recurrence of recommendations on breast and prostate cancer is highlighted as areas of focus (Table 3).

• **Detail of recommendations**

- Classification according to type of practice: those related to treatments in general and their different modalities (such as chemotherapy, radiotherapy) top the list, followed by diagnostic practices (Table 4).

- A crucial finding is that 93% of recommendations are based on evidence that has not been updated since 2018, underscoring the need for constant review and updating to ensure the validity of recommendations.

• **Consolidation and expert review**

- After eliminating duplicates and peer review, 30 unique recommendations were consolidated from the original 74.

- The expert review identified two inappropriate recommendations that were excluded: one in the process of revision and one obsolete and unlikely to be used. This emphasizes the importance of expert review in the final selection of recommendations.

**2. Prioritisation for Latin America**

• **Detailed profile of the expert panel**

- Active participation of 21 experts from 8 Latin American countries: Argentina, Brazil, Chile, Colombia, Honduras, Mexico, Paraguay and Peru.

- They represent a significant portion (about 70%) of the cancer patient population in the region.

- Diverse composition of the panel, including oncologists, epidemiologists and health policy experts (Table 1).

• **Consultation and categorisation phases**

- Grouping of recommendations into key categories: cancer screening, general cancer treatment, treatment for specific oncologic diseases and recommendations on palliative and radiotherapeutic treatment.

- Two rounds of consultation to prioritise recommendations and obtain an informed consensus.

Result: a final list of the ten most prioritised ‘inadvisable recommendations’ for the region was achieved, reflecting the international consensus adapted to the Latin American reality (Table 5).

This study was supported by a wide variety of source documents and recommendations made by other initiatives from different countries and continents and the multidisciplinary experience of Latin American experts, ensuring the validity and relevance of the recommendations in the regional context.

## Discussion

Clinical practice guidelines are a fundamental tool for adequate cancer care and they have evolved to the present time, where they not only propose what should be done but also what should NOT be done. The ‘CW’ methodology is an initiative of ABIM to avoid unnecessary medical tests, treatments and procedures. The mission of ‘CW’ is to promote conversations between physicians and patients by helping patients choose care that is supported by evidence, not duplicative of other tests or procedures already received, free from harm and truly necessary [1]. Our findings are expressed in the ten final proposed recommendations, which were identified after a literature review of previous lists of possible preventive, diagnostic and treatment interventions, selected from the opinion of a sufficient number of experts with vast experience in the field of Clinical Oncology. All the recommendations have more than 80% acceptance, so we believe that they will contribute to guide practice and improve the quality of oncologic care in the region. Our study selected the recommendations through a modified Delphi method, which allows the collection of expert opinion through a series of iterative questionnaires, with the aim of reaching a group consensus. Its main advantages include the low margin for error or bias, the contribution of each expert, and the simplicity of conducting it. Unlike other CW studies, the present one included an initial review of the existing literature to determine the most frequently used common response base. We also used two rounds of face-to-face meetings. One weakness or limitation we have found is that our experts – coming from the eight Latin American countries that account for 70% of oncology patients in the region as a whole – are all physicians with mostly clinical experience. We have not included representatives from other health sectors or patients, which may limit the value of some responses.

We have considered other studies similar to the present one and compared them with ours

- CW Africa*: Insights from the Front Lines of clinical care, published by* Rubagumya *et a*l [1]. This was a survey of oncologists belonging to the African Organisation for Research and Treatment of Cancer to review the agreement with the ten CW Africa recommendations published in 2020 in sub-Saharan African countries. The conclusion was that agreement with the recommendations was high, but that efforts to disseminate the recommendations need to be insisted upon. Forty-six percent of participants said they were unaware of the recommendations. Comparing CW Africa with CW Latin America, agreement was only 40% [1].
- CW for oncology in Brazil: ‘Ten recommendations to deliver evidence-based cancer care by de Moraes *et al* [10]. A multidisciplinary group of specialists from Brazil together with patient organisations used, like our work, a modified Delphi method and three teleconferences. Comparing this with CW Latin America, it is evident that the recommendations are the same or use a similar concept in seven recommendations, while three are different [10].
- CW Philippines: used a Clinical Consensus Committee of the Philippine Society of Medical Oncology according to the article ‘*CW Philippines: Ten Low-Value or Harmful Practices That Should Be Avoided in Cancer Care*’ by F. I. Ting et al. Here, comparing the two recommendations, it is evident that the same or similar concept is used in six recommendations, while four are different [2].
- CW Canada was developed by the societies of surgery, radiotherapy and oncology, together with the Canadian Partnership Against Cancer [11]. The group used a multidisciplinary process following the following criteria: (1) the size of the population for which the practice is relevant; (2) the frequency of use of the practice in Canada; (3) the cost of the practice; (4) evidence/degree of harm of the practice; and (5) the potential for change in the use of the practice. Our review shows that CW Canada was the only one to use these defined criteria, although the same criteria have possibly been included in the other evaluations, but we are unable to verify this. Comparing the Canadian and Latin American recommendations, there was only a 4/10 agreement. In general, those of Latin America were more related to the use of diagnostic and therapeutic procedures, and those of Canada with some inclination to actions in the health system [12].
- In the case of CW India, a multidisciplinary consensus was used to identify a list of harmful or low-value cancer practices that are frequently performed in that country. A working group convened by the National Cancer Grid of India included representatives from surgical, medical and radiation oncology. Each specialty had representation from both the public and private sectors. The working group included two national patient representatives and patient advocacy groups [10]. Comparing both CWs, there was complete agreement in 4/10 and NO agreement in 6/10 recommendations [13].

As we can see, the different CWs have used, on a common Delphi-like basis, variable methodologies, with participants sometimes multidisciplinary or sometimes only based on expert oncologists. However, most of them considered in their discussions the different CW published as a basis.

A clear strength of our study has been the Delphi-like methodology including face-to-face meetings along with virtual discussions.

An obvious weakness has been the limited participation of oncology specialists. Another disadvantage is that this technique does not produce a right or wrong answer and that the technique is based on opinion, so consensus does not necessarily mean that the answer is correct.

**Implication of the results and message**

- Methodological strengthening in future CW studies: Due to the weaknesses identified in the methodology of the present study, it is recommended that future CW be strengthened. To this end, the inclusion of multidisciplinary teams composed of health systems specialists and representatives of patient organisations is suggested. This broad participation will enrich the quality of future evaluations and ensure a comprehensive perspective in clinical decision-making.
- Contextualisation and regional relevance of the recommendations: It is evident that concordance or discordance in recommendations is closely linked to the particularities of health systems and variables specific to each country. This fact is evident when observing the low concordance in regions such as Africa, India and Canada (40%), compared to a higher concordance in Brazil (70%) and the Philippines (60%). Therefore, it is concluded that CW studies should be adapted and conducted taking into account the characteristics of each country or related regions. This adaptation is essential to avoid significant errors in the conclusions and to ensure the relevance of the recommendations in clinical practice.
- Rigorous methodologies to evaluate oncologic practices: Based on the results obtained and to ensure the scientific validity of future studies, the adoption of methodologies based on the Delphi method, either in its original or modified form, is strongly recommended. These methodologies allow for a more rigorous evaluation process, encouraging the participation and consensus of experts in the field of oncology. In addition, the use of simple surveys with less scientific rigor should be avoided to guarantee the quality and reliability of the evaluations in the context of CW.

## Conclusion

In conclusion, clinical practice guidelines have evolved to encompass not only what should be done but also what should be avoided, exemplified by initiatives like CW. CW, pioneered by ABIM, aims to foster evidence-based decision-making and patient-physician dialogue, emphasising care that is necessary, evidence-supported, and devoid of harm.

Our study contributes to this mission by proposing ten recommendations for oncologic care, derived through a rigorous process involving literature review, expert consensus, and a modified Delphi method. These recommendations, with over 80% acceptance, are poised to enhance the quality of cancer care in our region.

However, while our methodology boasts advantages such as low error margins and expert contribution, we acknowledge limitations. Primarily, our expert panel comprised physicians with clinical experience, potentially excluding valuable perspectives from other healthcare sectors and patients. This gap suggests avenues for future research to broaden stakeholder inclusion, ensuring guidelines reflect a comprehensive understanding of oncologic care needs.

## Conflicts of interest

The authors declare that they have no conflicts of interest.

## Funding

This project was made possible by an unrestricted grant from Bristol Myers Squibb (BMS).

## Figures and Tables

**Table 1.** Comprehensive review of ‘CW’ and other oncology divestment initiatives.

Stage of the continuum of care	ID	Proposed initiative	Recommendation	Condition
Prevention and screening	1	-Divestment Argentina 2021 -CW Australia (Royal College of Pathologists of Australasia) -CW Australia (Medical Oncology Group of Australia)	Do not test for serum tumour markers except to evaluate or monitor a cancer known to produce these markers.	Cancer
	2	Divestment Argentina 2021	In relation to breast screening, it is suggested NOT to perform the following practices: 1. Counselling for breast self-examination and clinical breast examination IN REPLACE OF MAMMOGRAPHY, which is the method of choice for breast cancer screening. 2. Mammography in women under 40 years of age with no risk factors; between 40 and 49 years of age, the decision to perform mammography depends on the value the woman places on the benefits and risks of screening. Breast magnetic resonance imaging (MRI) with and without intravenous contrast in women with no risk factors, or with an average – less than 15% lifetime risk of breast cancer (in this group, digital tomosynthesis is appropriate and ultrasound may be appropriate).	Breast cancer
	3	-CW – ASCO – USA -CW Australia (Royal College of Pathologists of Australasia) -CW – American College of Preventive Medicine	Do not routinely perform PSA testing for prostate cancer screening in men without symptoms of the disease.	Prostate cancer
	4	CW Canada – Updated July 2021	Do not perform routine cancer screening, or surveillance for a new primary cancer, in most patients with metastatic disease.	Metastatic cancer
	5	-CW India 2019 -CW Africa 2020 -CW Australia (Medical Oncology Group of Australia)	Avoid testing (biomarkers and imaging) for recurrent cancer in previously treated asymptomatic patients, unless there is evidence that early detection of recurrence can improve survival or quality of life; including avoiding tests of Surveillance (biomarkers) or imaging (PET, CT and radionuclide bone scintigraphy) for asymptomatic individuals who have been treated for breast cancer with curative intent.	Recurrent Cancer
Diagnosis	6	CW – ASCO – USA	Avoid using PET or PET-CT to monitor response to palliative chemotherapy or as part of routine follow-up care to detect cancer recurrence in asymptomatic patients who have completed initial treatment to eliminate the cancer unless there is high-level evidence that such imaging will change the outcome.	Recurrent cancer
	7	CW Canada – Updated July 2021	Do not perform routine colonoscopic surveillance every year in patients after their colon cancer surgery; instead, the frequency should be based on previous colonoscopy findings and corresponding guidelines.	Colon cancer
	8	CW – ASCO – USA	Do not perform PET, CT and radionuclide bone scans in the staging of early breast cancer with low risk of metastasis.	Breast cancer
	9	CW – ASCO – USA	Do not perform surveillance testing (biomarkers) or imaging (PET, CT and radionuclide bone scans) for asymptomatic individuals who have been treated for breast cancer with curative intent.	Breast cancer
	10	-CW Italy – Federazione delle Associazioni dei Dirigenti Ospedalieri Internisti (FADOI) -CW – American College of Preventive Medicine -CW Italy – Associazione Italiana di Medicina Nucleare e Imaging Molecolare (AIMN)/Italian Association of Nuclear Medicine -CW Italy – Hospital S. Croce e Carle Cuneo -CW- Society of Nuclear Medicine and Molecular Imaging	Do not perform PET (positron emission tomography) or CT (computed tomography) for cancer screening in healthy subjects.	Cancer
	11	-CW -Commission on Cancer -CW Italy – Associazione Italiana di Radioterapia Oncologica (AIRO) -CW -American Urological Association -CW Holland -CW Italy – Hospital S. Croce e Carle Cuneo -CW -American Urological Association	PET, CT or bone scintigraphy is not recommended for staging prostate cancer with low risk of metastasis in patients receiving radical radiotherapy, except under clinical research conditions.	Prostate Cancer
	12	-CW Italy – Associazione Italiana di Medicina Nucleare e Imaging Molecolare (AIMN)/Italian Association of Nuclear Medicine -CW Italy – Hospital S. Croce e Carle Cuneo -CW Italy – Hospital S. Croce e Carle Cuneo	Do not perform lymphoscintigraphy and radio-guided sentinel lymph node biopsy in patients with melanoma of the skin with a thickness of less than 0.75 mm, non-ulcerated, and a value of mitoses <1/mm^2^.	Melanoma
Treatment	13	-CW India 2019 -CW Canada – Updated July 2021	Do not provide care (e.g., follow-up) in a high-cost setting (e.g., hospitalisation, cancer center) that could be provided just as effectively in a lower-cost setting (e.g., primary care).	Cancer
	14	-CW – ASCO – USA -CW India 2019	Do not use white blood cell stimulating factors for prevention of febrile neutropenia in patients with less than 20% risk of this complication.	Cancer
	15	-CW – ASCO – USA	Do not give antiemetics to patients starting a chemotherapy regimen that has a low to moderate risk of causing nausea and vomiting.	Cancer
	16	-CW Africa 2020	Do not use surgery as initial treatment without considering pre-surgical systemic therapy (neoadjuvant) and/or radiation for certain types and stages of cancer where it has been shown to be effective in improving local cancer control, quality of life and/or survival.	Cancer
	17	-CW India 2019	Do not use advanced radiotherapy techniques when conventional radiotherapy can be equally effective.	Cancer
	18	-CW Australia (Royal Australian and New Zealand College of Radiologists) -CW- American Society for Radiation Oncology	Do not routinely add adjuvant whole brain radiotherapy to stereotactic radiosurgery for limited brain metastases.	Brain Cancer
	19	-CW Africa 2020	Do not perform surgery to remove a breast lump without histological confirmation of malignancy unless a needle biopsy cannot be performed.	Breast cancer
	20	-CW Africa 2020 -CW – ASCO – USA -CW- American Society of Clinical Oncology	Do not use combination (multiple drug) chemotherapy instead of single (single) drug chemotherapy when treating a person for metastatic breast cancer unless the patient needs a rapid response to relieve tumour-related symptoms.	Breast cancer
	21	-CW India 2019 -CW Canada – Updated July 2021 -CW Africa 2020	Do not initiate whole-breast radiation therapy in 25 fractions as part of breast-conserving therapy in women aged 50 years or older with early-stage invasive disease breast cancer without considering more abbreviated treatment schedules.	Breast cancer
	22	Divestment Argentina 2021	Regarding treatment with high-cost drugs in women with breast cancer: the association of trastuzumab plus bevacizumab as first-line treatment is not recommended; the use of Bevacizumab with either taxanes or capecitabine as first-line treatment is not recommended in women with metastatic disease; the use of the combination Lapatinib plus chemotherapy as first-line treatment in patients with HER2-positive metastatic or locally recurrent unresectable breast cancer is not recommended.	Breast cancer
	23	-CW Africa 2020 -CW Canada – Updated July 2021 -CW (Royal Australian and New Zealand College of Radiologists) -CW -American Urological Association	Do not treat prostate with clinically localised low-risk cancer Cancer (e.g., Gleason score <7, prostate-specific antigen < 10.0 ng/mL and tumour stage T2) without discussing active surveillance as part of the shared decision-making process	Prostate cancer
	24	Divestment Argentina 2021	The use of Erlotinib, Gefitinib and Afatinib in patients with metastatic Non-Small Cell Lung Cancer in the absence of EGFR mutations is discouraged.	Non-small cell lung cancer
	25	Divestment Argentina 2021	Trabectidine associated with liposomal doxorubicin in patients with epithelial ovarian cancer with recurrence between 6 and 12 months after a platinum-based first line.	Epithelial ovarian cancer
	26	-CW Canada – Updated July 2021 -CW Australia (Medical Oncology Group of Australia)	Do not routinely use extensive locoregional therapy in most cancer situations where there is metastatic disease and minimal symptoms attributable to the primary tumour (e.g., colorectal cancer).	Metastatic cancer
	27	CW India 2019	Do not treat patients with advanced metastatic cancer in intensive care units unless there is a reversible event.	Advanced metastatic cancer
	28	CW Commission on Cancer	Do not initiate cancer treatment without defining the extent of the cancer (through clinical staging) and discussing with the patient the intention of treatment.	Cancer
Rehabilitation and Palliative Care	29	-Divestment Argentina 2021 -CW India 2019 -CW Africa 2020 -CW Canada – Updated July 2021 -CW Australia (Medical Oncology Group of Australia) -CW Italy – Collegio Italiano Primari di Oncologia Medica Green Oncology -CW – ASCO – USA	Do not use cancer-directed therapy in patients with solid tumours who meet the following conditions: low-performance status (3 or 4); no benefit from previous evidence-based interventions (disease progression after 2 or 3 lines of treatment); not eligible for inclusion in clinical trials; no strong evidence available to support the clinical value of any additional anti-cancer therapy. Instead, prioritise palliative treatment to relieve symptoms.	Advanced cancer
	30	-CW- American Academy of Hospice and Palliative Medicine -CW- American Academy of Hospice and Palliative Medicine	It is not recommended to perform more than a fraction of palliative radiation for uncomplicated painful bone metastasis.	Metastatic cancer

**Table 2.** Panel of experts.

Expert	Country	Specialty	Affiliation
Julia Ismael, MD	Argentina, CABA	Global Health-Oncologist-Former INC Director	GEDYT S.A.
Eugenia Esandi, Md	Argentina, CABA	Epidemiology-Methodology	National Academy of Medicine
Ernesto Gil Deza	Argentina, CABA	Oncologist	Henry Moore Institute
Silvana Rompato	Argentina, Formosa	Oncologist	Argentine Association of Clinical Oncology
Tatiana Vidaurre	Peru	Salud Global-Mastologa-Global Health- Mastologa- Ex Director INEN Perú	National Institute of Neoplastic Diseases, (INEN) Peru
Daniel Lewi	Argentina, CABA	Oncologist	Public and private practice.
Cinthia Gauna	Paraguay	Oncologist. INCAN-IPS-MIGONE	Paraguayan Society of Medical Oncology
Gerardo Arroyo	Argentina, Salta	Oncologist	CeDIT
Francisco Gutiérrez-Delgado	Mexico	Oncologist	ELO-CEPREC
Carlos Castro	Colombia	Surgeon- Former Director INC Colombia	Colombian League Against Cancer
Angela Solano	Argentina	Director,Argentine Node of the Human Variome Project	Center for Medical Education and Clinical Research - HUSS
Bettina Müller	Chile	Oncology – Clinical Research	Chilean Cooperative Research Oncology Group
Luiz Santini	Brazil	Surgeon- Former Director INC Brazil	Associate Researcher FIOCRUZ
Jorge Puyol	Argentina	Oncologist	Argentine Cancer Society
Gabriela Quintanilla	Argentina, Santa Fe	Oncologist – Former Director Provincial Cancer Agency	
Claudia Enrique	Argentina, Entre Ríos	Mastologist – Director of the Provincial Cancer Agency	Provincial Cancer Institute, Entre Ríos
Raul Murillo	Colombia	Epidemiologist- Former Director INC Colombia	HUSI Xaverian Oncology Center
Suyapa Bejarano	Honduras	Oncologist	League Against Cancer
Sergio Becerra	Chile	Oncologist. Former Chief of Comprehensive Cancer Management.	CARE Foundation
Alicia Pomata	Paraguay	Mastologist- C-Can Paraguay	National Cancer Control Program
Karin Kopitowski, MD	Argentina,	Internal Medicine	Argentine Society of Family Medicine. Italian Hospital
Eduardo Cazap, MD	Argentina, CABA	Former President UICC	SLACOM- eCancer

**Table 3.** ‘Do not do’ recommendations by condition.

Condition	Quantity Rec NH	Percentage
Cancer	28	38
Metastatic/Advanced cancer	16	22
Prostate cancer	13	18
Breast cancer	9	12
Melanoma	3	4
Brain cancer	2	3
Colon cancer	1	1
Non-small cell lung cancer	1	1
Epithelial ovarian cancer	1	1
Total	74	100

Source: Own elaboration

**Table 4.** ‘Do not do’ recommendations by type of practice.

Practice	Quantity Rec NH	Percentage
Diagnosis	19	26
Treatment	16	22
Treatment chemotherapy	14	19
Screening	12	16
Radiant treatment	9	12
Palliative xare	4	5
Total	74	100

Source: Own elaboration

**Table 5.** Top 10 ‘Choosing Wisely‘ recommendations in oncology in Latin America.

1	Do not use cancer-directed therapy in patients with solid tumours with low-performance status (3 or 4); no benefit from previous interventions, and not eligible for inclusion in clinical trials. Prioritise palliative treatment to relieve symptoms.
2	Do not start cancer treatment without defining the extent of the disease (staging) and discussing with the patient the intention of treatment.
3	Do not test for tumour markers except to assess or monitor active disease.
4	Do not perform PET (positron emission tomography) or CT (computed tomography) for cancer screening in healthy subjects.
5	Do not perform routine colonoscopic surveillance yearly in patients after colon cancer surgery; frequency should be based on previous colonoscopy findings and guidelines.
6	The use of PET/CT is not recommended for the follow-up of patients undergoing palliative cancer treatment. It should not be routinely used to detect possible disease recurrence in patients who have completed cancer treatment, unless clinically suspected and/or suspected by imaging.
7	In the absence of EGFR mutations in non-small cell lung cancer, the use of tyrosine kinase inhibitors (Anti-EGFR) is not recommended.
8	Do not treat low-risk localised prostate cancer (e.g., Gleason score < 7, prostate-specific antigen < 10.0 ng/mL, and tumour stage T2) without discussing active surveillance as part of the decision-making process.
9	Avoid biomarker testing and imaging for recurrent cancer in previously treated asymptomatic patients unless there is evidence that early detection of recurrence may improve survival or quality of life; including avoiding biomarkers or imaging in asymptomatic patients treated for breast cancer with curative intent.
10	Do not routinely apply adjunctive holocranial radiotherapy when the patient has undergone stereotactic radiotherapy.
